# Low-dose tolvaptan PK/PD: comparison of patients with hyponatremia due to syndrome of inappropriate antidiuretic hormone secretion to healthy adults

**DOI:** 10.1007/s00228-017-2302-7

**Published:** 2017-08-12

**Authors:** Susan E. Shoaf, Patricia Bricmont, Ann Dandurand

**Affiliations:** 1 Otsuka Pharmaceutical Development & Commercialization, Inc., Princeton, NJ USA; 2 2440 Research Blvd, Rockville, MD 20850 USA

**Keywords:** Tolvaptan, Pharmacokinetics, Pharmacodynamics, Syndrome of inappropriate diuretic hormone (SIADH), Healthy subjects

## Abstract

**Purpose:**

Tolvaptan (TLV) is indicated to treat hyponatremia due to syndrome of inappropriate diuretic hormone (SIADH) in Europe. Treatment is to be initiated at 15 mg QD but post-approval reporting indicates increasing use of 7.5 mg as the starting dose. Physicians believe 7.5 mg is effective and has a lower incidence of overly rapid correction of serum sodium.

**Methods:**

Single TLV doses of 3.75, 7.5, and 15 mg were administered to 14 healthy adults in a crossover design and to 29 subjects ≥18 years with SIADH and serum sodium between 120 and 133 mmol/L in a parallel-group design. Pharmacodynamics and TLV plasma concentrations were assessed for 24 h post-dose.

**Results:**

In SIADH subjects, corrections of serum sodium (Δ of ≥8 mmol/L in the first 8 h or ≥12 mmol/L in the first 24 h) were observed in one, one, and two subjects in the 3.75-, 7.5-, and 15-mg dose groups. Fluid balance (FB) for 0–6 h post-dose was correlated (*r* ^2^ = 0.37) with maximum increases in serum sodium; subjects with large corrections had large (~1 L) negative FB. Compared to healthy adults, subjects with SIADH did not drink in response to their negative FB and had larger increases in serum sodium at 24 h. Median time of maximum increase in healthy adults was 6 h, with no rapid corrections, and FB was near 0 mL by 24 h.

**Conclusion:**

Starting titration with 7.5 mg TLV will not eliminate the risk of rapid corrections in serum sodium. Monitoring FB may indicate that a subject is at risk for over correction.

## Introduction

Tolvaptan is approved in the USA for the treatment of adult patients with clinically significant hyponatremia associated with euvolemic and hypervolemic states [1]. In Europe, tolvaptan is approved for the treatment of adult patients with hyponatremia secondary to syndrome of inappropriate antidiuretic hormone secretion (SIADH) [2].

Tolvaptan acts as an orally available selective antagonist of the vasopressin V2-receptor [3]. By blocking the activity of vasopressin (antidiuretic hormone) on renal collecting tubules, the drug prevents the reabsorption of water, which leads to an increase in free water clearance (aquaresis) and a resulting increase in serum sodium concentrations. These pharmacologic consequences are the basis of its beneficial effects in patients with hyponatremia and/or edema. Tolvaptan treatment is to be initiated at 15 mg once daily with the dose increased to a maximum of 60 mg once daily as tolerated to achieve the desired level of serum sodium.

In clinical trials investigating treatment of hyponatremia, tolvaptan was well tolerated. The most common treatment-emergent adverse events associated with tolvaptan use have been consistent with its mode of action to promote free water clearance. Thus, in the pivotal efficacy (SALT) clinical trials, which enrolled hyponatremic patients with chronic heart failure, cirrhosis, or SIADH, the most common adverse events were thirst (16%), dry mouth (13%), and pollakiuria or polyuria (11%) [4]. In a subanalysis focusing on the subset of subjects with SIADH, thirst and dry mouth occurred in 18 and 16% of subjects, respectively, while increased urinary frequency occurred in 10% [5]. As with any therapy for hyponatremia, the primary safety concern is overly rapid correction of serum sodium concentrations (e.g., >12 mmol/L/24 h or >18 mmol/L/48 h), which can lead to osmotic demyelination of neurons (1) which may result in dysarthria, mutism, dysphagia, lethargy, affective changes, spastic quadriparesis, seizures, coma, and death. In the SIADH subset of the SALT trials, overly rapid correction of serum sodium after the first dose of tolvaptan occurred in 5.9% (3/51) of patients. All three of these patients had moderate hyponatremia at baseline (serum [Na^+^] < 130 mmol/L) [5].

In post-marketing assessments in Europe of tolvaptan use to treat hyponatremia, it has been reported that physicians, on a patient-by-patient case basis, appear to be splitting/crushing tolvaptan tablets to create a 7.5-mg starting dose in the belief that a lower starting dose may reduce the risk of overly rapid correction of serum sodium (data on file). No pharmacokinetic (PK)/pharmacodynamic (PD) trials had been conducted in SIADH patients with hyponatremia, so a clinical trial to investigate the initial response of serum sodium concentrations following doses of tolvaptan as single oral tablets of 15, 7.5, and 3.75 mg was performed (NCT02009878).

Investigation of tolvaptan for treatment of hyponatremia in pediatric subjects is ongoing. Prior to the start of this program, a phase 1 healthy-adult subject trial was conducted to compare the relative bioavailability of a suspension formulation to the 15-mg tablet, as well as to determine the relative bioavailability of tolvaptan following dosing with 7.5- and 3.75-mg tablets. The PK and PD assessment schedules following each dose were almost identical to those used in the SIADH patient trial, making it possible to directly compare the two populations.

In healthy adults, the PK and PD of single oral tolvaptan doses ranging from 15 to 480 mg have been previously reported [6–10]. The terminal-phase elimination half-life (*t*_1/2,z_) of tolvaptan increases with increasing dose, with mean values around 3 h for a 15-mg dose and 12 h for 120- to 480-mg doses. Tolvaptan is mostly absorbed from the upper gastrointestinal (GI) tract at lower doses, and the decline of the terminal portion of the concentration curve therefore primarily reflects elimination processes. With increasing dose, tolvaptan continues to be absorbed from the GI tract, such that the rate of decline of the terminal portion of the concentration curve is reflective of both absorption and elimination. The limited early absorption of tolvaptan is exemplified by the fact that maximum (peak) plasma concentration (*C*_max_) values show less than dose proportional increases from 30 to 240 mg and then plateau at doses from 240 to 480 mg. Despite the changes in tolvaptan absorption, area under the concentration-time curve (AUC) for tolvaptan following single oral doses increases proportionally with increasing dose, and apparent clearance of drug from plasma after extravascular administration (CL/F) values is unchanged for single doses from 30 to 480 mg. Tolvaptan concentrations do not accumulate following once daily dosing, and its PK parameters are not affected by age, gender, or race.

## Methods

### Ethics

The studies in this report were conducted in compliance with International Conference on Harmonisation Good Clinical Practice guidelines for conducting, recording, and reporting clinical trials, as well as for archiving essential documents. Consistent with ethical principles for the protection of human research subjects, no trial procedures were performed on trial candidates until written consent had been obtained. The informed consent form, protocol, and amendments for the study were submitted to and approved by the institutional review board or independent ethics committee at participating sites.

### Trial design: Trial 202

Trial 156-12-202, referred to in this report as Trial 202, was a single-center, open-label, four-period, randomized crossover trial in healthy adults conducted in the USA. Eligible subjects were 18 to 45 years of age and had a body mass index (BMI) of 19.0 to 32.0 kg/m^2^ and no clinically significant abnormalities in past medical history. Subjects were excluded if they had consumed grapefruit or grapefruit juice, Seville oranges or Seville orange juice, food or beverages containing methylxanthines, or alcohol within 72 h prior to study drug dosing. Use of prescription, over-the-counter or herbal medications, vitamin supplements, antibiotics, tobacco products, and substances known to stimulate hepatic microsomal enzymes was also prohibited.

#### Periods 1 and 2

The primary objective of the study was to compare the bioavailability of tolvaptan as an oral suspension relative to the currently approved formulation. On day 1, eligible subjects were randomized 1:1 to receive a single oral dose of 15-mg tolvaptan administered either as a suspension or a tablet in the morning. On day 3, subjects crossed over to receive the opposite treatment.

#### Periods 3 and 4

The secondary objective of the study was to determine the pharmacokinetics and pharmacodynamics of oral tolvaptan at 3.75-, 7.5-, and 15-mg dosage. Thus, on day 5 and day 7, all subjects received a single oral 7.5- and 3.75-mg tablet of tolvaptan, respectively, in the morning.

Doses were administered following an overnight fast of at least 10 h. The 15-, 7.5-, and 3.75-mg tablet doses were administered with dose-proportional amounts of still, room temperature water, 240, 120, and 60 mL, respectively. After 2 h post-dose, subjects were to have free access to water and were instructed to drink in response to thirst. A safety follow-up telephone contact was performed 7 days after the last dose was administered.

### Trial design: Trial 203

Trial 156-12-203 (NCT02009878), referred to in this report as Trial 203, was a multinational, multicenter, randomized, parallel-group, double-blind trial in adults with a diagnosis of SIADH. Eligible subjects were 18 years of age or older with a BMI ≤ 32 kg/m^2^. Subjects also had persistent euvolemic hyponatremia (serum sodium measurements of 120–133 mmol/L on three separate assessments (occurring during the screening period, on day −1 and on day 1) and relatively intact renal function (an estimated glomerular filtration rate of ≥60 mL/min/1.73 m^2^ using the CKD-EPI formula [11]. Key exclusion criteria included concomitant anti-hyponatremia therapy, severe hepatic impairment, ingestion of CYP3A4 inhibitors or inducers, and consumption of grapefruit or grapefruit juice, Seville oranges or Seville orange juice, food or beverages containing methylxanthines, and/or alcohol within 72 h prior to study drug dosing. Subjects with medication-induced SIADH were eligible if the etiologic agent had been stable for at least 3 months. The primary objective of the trial was to determine the maximal increase and time of maximal increase in serum sodium concentrations following single oral tolvaptan doses of 3.75, 7.5, and 15 mg in subjects with SIADH.

Eligible subjects were randomized 1:1:1 on day 1 to receive a single dose of oral tolvaptan at 3.75, 7.5, or 15 mg. Doses were administered using 240 mL of still, room temperature water in the morning following an overnight fast of at least 10 h. After 2 h post-dose, subjects were to have free access to water and were instructed to drink in response to thirst. A safety follow-up telephone contact was performed 7 days after the last dose was administered.

### PK and PD sampling

Blood samples (4 mL, heparin) for determination of plasma concentrations of tolvaptan were collected predose and at 1, 2, 3, 4, 6, 8, 12, 16, and 24 h post-dose.

Blood samples (5 mL, serum separator tube) for PD assessments were collected for each dose at predose and at 2, 4, 6, 8 (Trial 203 only), 12, and 24 h post-dose. Urine samples were collected from 0 to 2, 2 to 4, 4 to 6, 6 to 8, 8 to 12, 12 to 16, and 16 to 24 h (Trial 202) or 12 to 24 h (Trial 203) post-dose. Total fluid intake was measured from 0 to 6, 6 to 12, and 12 to 24 h post-dose.

On day −1, subjects were in the clinic to have baseline PD assessments. For Trial 202, baseline assessments were identical to the timing of post-dose assessments, including drinking 240 mL water at time 0 to mimic dosing. For Trial 203, urine and fluid intake assessments were identical to post-dose assessments but assessments in serum were limited to predose and 12 h.

### Bioanalytical methods

Plasma concentrations of tolvaptan were analyzed using reversed-phase high-performance liquid chromatography with tandem mass spectrometric detection as described previously [6]. Briefly, tolvaptan and an internal standard were extracted from 250 μL heparinized plasma using solid phase extraction. Calibration standards prepared in plasma and extracted along with samples were used to quantitate the concentrations by weighted (1/*x* ^2^) linear regression of peak area ratios of analyte-to-internal standard. The method was revalidated for concentrations ranging from 1.00 to 200 ng/mL using 25 μL plasma. Quality control samples were evaluated during validation to assess performance of the method and resulted in a percent coefficient of variation of ≤7.6% and relative error between −6.0 and 2.7%. Plasma analysis was performed at ICON Development Solutions (Whitesboro, NY).

Serum and urine osmolality, sodium, potassium, and creatinine were determined at the local clinical chemistry laboratory (Trial 202) or the central clinical chemistry laboratory (Trial 203) according to standard operating procedures.

### PK and PD endpoints

#### Trial 202

The primary PK endpoints for this trial were *C*_max_, AUC calculated to the last observable concentration at time *t* (AUC_*t*_), and AUC from time 0 to infinity (AUC_∞_) for tolvaptan in plasma. The secondary PK endpoints were *t*_max_, *t*_1/2,z_, and CL/F for tolvaptan in plasma. The secondary PD endpoints were urine volume, fluid intake, and fluid balance for 0–6, 0–12, and 0–24 h post-dose; free water clearance each urine collection interval; and sodium, potassium, creatinine, and osmolality serum and urine concentrations. Other PD outcomes included maximal change from baseline in serum sodium and time of maximal change from baseline.

#### Trial 203

The primary PD endpoints were the maximal increase from baseline and time of maximal increase from baseline in serum sodium concentration following tolvaptan administration. The secondary PK endpoints for this trial were the same PK parameters as determined in Trial 202. The secondary PD endpoints were the same PD endpoints as listed for Trial 202.

### Pharmacokinetic analysis

#### Trial 202

Plasma PK endpoints for tolvaptan were based on actual blood-sample times in all calculations. Values of *C*_max_ and *t*_max_ were determined directly from the observed data. Values of AUC_*t*_ were estimated using the linear trapezoidal rule. The terminal-phase elimination rate constant (*λ*_z_) was estimated by a log-linear regression of at least three non-zero concentrations. The *t*_1/2,z_ was determined as (ln2)/*λ*_z_. The value of CL/F was determined as dose/AUC_∞_/body weight. Plasma PK parameter calculations were performed using Phoenix WinNonlin®, version 6.2.1 (Pharsight Corporation, Mountain View, CA).

#### Trial 203

Plasma PK endpoints were determined as for Trial 202, with calculations performed using Phoenix WinNonlin® (version 6.4).

### Pharmacodynamic analysis

Fluid balance was determined as fluid intake minus urine output. Fluid intake included water used for dosing and foods with high water content (e.g., soup).

Cumulative urine was not determined if urine volume for an interval was missing; volumes of 0 were not considered missing. Subjects were asked to void prior to the end of each collection interval with a urine sample at 24 h required. Interim voids were kept refrigerated and, if available, were pooled with the final void of the interval, the volume determined, and a sample taken for sodium, potassium, creatinine, and osmolality determination.

Free water clearance was determined as urine excretion rate minus osmolar clearance. Osmolar clearance was determined by standard methods with average osmolality in serum determined by averaging the concentrations at the beginning and end of the urine collection interval.

### Statistical analysis

Pharmacodynamic endpoints and changes from baseline were summarized using descriptive statistics by time point or collection interval and tolvaptan dose using SAS version 9.1 (Trial 202) or version 9.4 (Trial 203) (SAS Institute, Cary, NC). PK descriptive statistics were determined using S-Plus, version 8.2 (TIBCO Software, Inc., Boston, MA). Slopes and 95% confidence intervals (CIs) of plots of individual subject log dose versus Log *C*_max_ or Log AUC_∞_ were determined in Sigma Plot, version 12.5 (Systat Software, Inc., San Jose, CA) as were correlations between maximal increases in serum sodium, fluid balance, and urine volume.

## Results

Table 1 summarizes demographics and baseline characteristics of subjects who had measurable tolvaptan plasma concentrations. For Trial 202, 14 healthy adult subjects were enrolled and all completed the trial. For Trial 203, 30 subjects were randomized, 10 to 3.75, 11 to 7.5, and 9 to 15 mg. In the 7.5-mg group, one subject was withdrawn prior to dosing after failing the alcohol screening test. In the 15-mg dose group, all subjects were reportedly dosed, but one subject had no measurable tolvaptan plasma concentrations, and the PD profile was consistent with no tolvaptan action; this subject was excluded from the PK and PD analyses. One subject (15-mg dose group) was missing the predose serum sodium concentration on day 1 so maximal increase in serum sodium could not be determined.

**Table 1:** Baseline characteristics

**Parameter**	Healthy adults	SIADH patients		
	**(** ***N*** **= 14)**	**3.75 mg (** ***N*** **= 10)**	**7.5 mg (** ***N*** **= 10)**	**15 mg (** ***N*** **= 8)**
Age (year)	32.3 (3.7)	68.5 (8.7)	70.2 (9.9)	59.0 (16.4)
Weight (kg)	83.6 (8.0)	66.9 (20.5)	60.8 (13.8)	65.4 (16.0)
Body mass index (kg/m^2^)	26.3 (2.7)	23.7 (5.3)	22.4 (4.0)	23.4 (4.7)
Race (*n*, %)				
Caucasian	4 (28.6)	10 (100)	10 (100)	8 (100)
Black or African American	8 (57.1)	0	0	0
Native Hawaiian	1 (7.1)	0	0	0
Other	1 (7.1)	0	0	0
Ethnicity (*n*, %)				
Hispanic or Latino Not Hispanic or Latino	4 (57.1) 10 (42.9)	– –	– –	– –
Gender (*n*, %)				
Male Female	8 (57.1) 6 (42.9)	4 (40.0) 6 (60.0)	3 (30.0) 7 (70.0)	3 (37.5) 5 (62.5)
Known underlying etiology of SIADH (*n*, %)				
Tumors CNS disorder Drug induced	NA	3 (30.0) 1 (10.0) 0 (0.0)	2 (20.0) 0 (0.0) 0 (0.0)	2 (22.2) 2 (22.2) 2 (11.1)
eGFR_CKD-EPI_ (mL/min/1.73 m^2^)	111 (14.1)	91.4 (18.8)	88.5 (11.0)	96.4 (19.1)
Serum sodium (mmol/L)^a^	139.3 (1.9)	130.9 (5.4)	132.2 (2.7)	131.5 (4.1)

Values are mean (standard deviation) unless otherwise noted;

*N* = number of subjects included in PK/PD analyses

^a^Predose day 0

No subjects in Trial 202 received concomitant medications while taking tolvaptan. Over 90% of subjects in Trial 203 total received one or more concomitant medications while taking tolvaptan. The most common (≥15% of subjects overall) classes of concomitant medications used while taking tolvaptan were as follows: drugs for acid-related disorders (41.4%), antithrombotic agents (34.5%), beta blocking agents (34.5%), psycholeptics (27.6%), agents acting on the renin-angiotensin system (31.0%), lipid-modifying agents (24.1%), analgesics (20.7%), calcium channel blockers (17.2%), cardiac therapy (17.2%), and vitamins (17.2%). The most common (≥10% of subjects overall) individual concomitant medications taken while on study drug included omeprazole (24.1%), pantoprazole (20.7%), bisprolol (20.7%). acetylsalicylic acid (17.2%), colecalciferol (13.8%), atorvastatin (13.8%), warfarin (10.3%), amlodipine (10.3%), mesalazine (10.3%), simvastatin (10.3%), potassium chloride (10.3%), metformin (10.3%), metamizole (10.3%), and nebivolol (10.3%). None of these medications would be expected to have PK or PD interactions with tolvaptan.

**Fig. 1:**
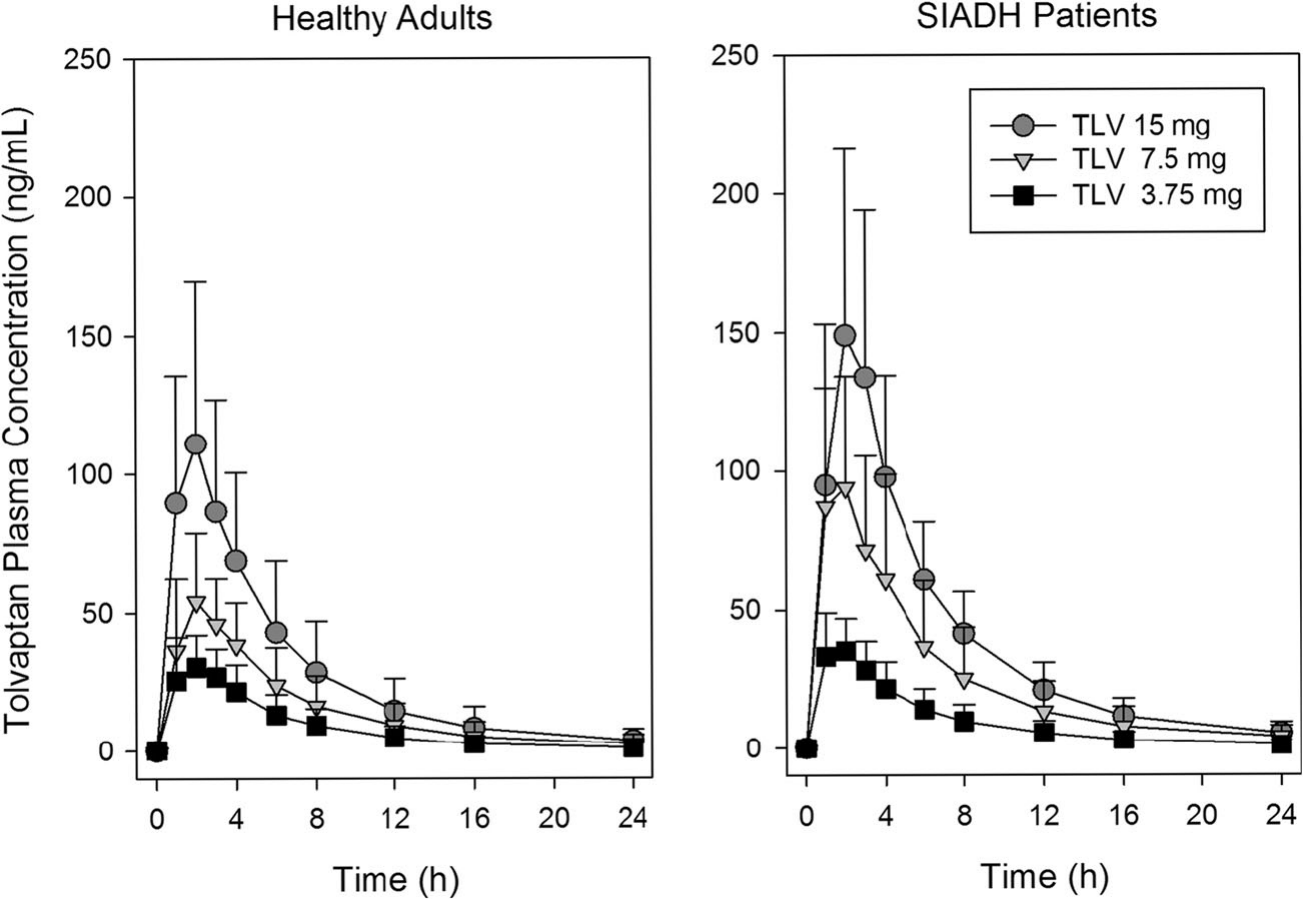
Mean (SD) tolvaptan plasma concentrations following single oral doses of tolvaptan to healthy adults and SIADH patients with hyponatremia

Plots of mean (SD) tolvaptan plasma concentrations for 24 h post-dose are presented in Fig. 1. Results of the pharmacokinetic analysis are presented in Table 2. Concentrations following 7.5- and 15-mg doses were slightly higher in SIADH subjects when compared to healthy adults. Concentrations increased dose proportionally in SIADH patients with the slope of the log *C*_max_ and log AUC versus log dose plots equal to 1.02 for both parameters. Concentrations increased less than dose proportionally in healthy adults, as the slopes for the log-log plots were 0.82 for both parameters; concentrations for the 3.75-mg dose group were higher than expected when compared to the higher doses, which showed dose proportional increases in concentrations. In both trials, there was large overlap in observed tolvaptan concentrations when the 7.5-mg dose was compared to either the 3.75- or 15-mg doses, as the percent coefficients of variation for *C*_max_ and AUC ranged from 33 to 57%. As a sensitive CYP3A4 substrate, this degree of variability in pharmacokinetic parameters is not unexpected.

**Table 2:** Mean (SD) tolvaptan pharmacokinetic parameters following single oral doses of tolvaptan to healthy adults and SIADH patients with hyponatremia

**Parameter**	**Healthy adults**			**SIADH patients**		
	**3.75 mg (** ***N*** **= 14)**	**7.5 mg (** ***N*** **= 14)**	**15 mg (** ***N*** **= 14)**	**3.75 mg (** ***N*** **= 10)**	**7.5 mg (** ***N*** **= 10)**	**15 mg (** ***N*** **= 8)**
*C*_max_ (ng/mL)	35.6 (13.2)	57.8 (25.4)	119 (56.5)	37.7 (12.6)	107 (48.9)	157 (68.1)
*t*_max_ (h)^a^	2.00 (1.00–4.03)	2.00 (1.00–3.00)	2.00 (1.00–4.05)	1.50 (0.95–4.00)	2.00 (1.00–4.00)	2.00 (0.97–3.00)
AUC_∞_ (ng · h/mL)	222 (124)	398 (235)	728 (415)	244 (90.7)	655 (373)	1000 (361)
*t*_1/2,z_ (h)	4.3 (1.8)	5.2 (2.0)	5.8 (2.0)	4.6 (2.3)	6.0 (2.5)	5.9 (2.1)
CL/F (mL/min/kg)	4.81 (2.66)	5.46 (2.96)	5.90 (3.32)	5.00 (3.09)	5.74 (6.11)	4.91 (3.44)

^a^Values are median (minimum-maximum)

**Fig. 2:**
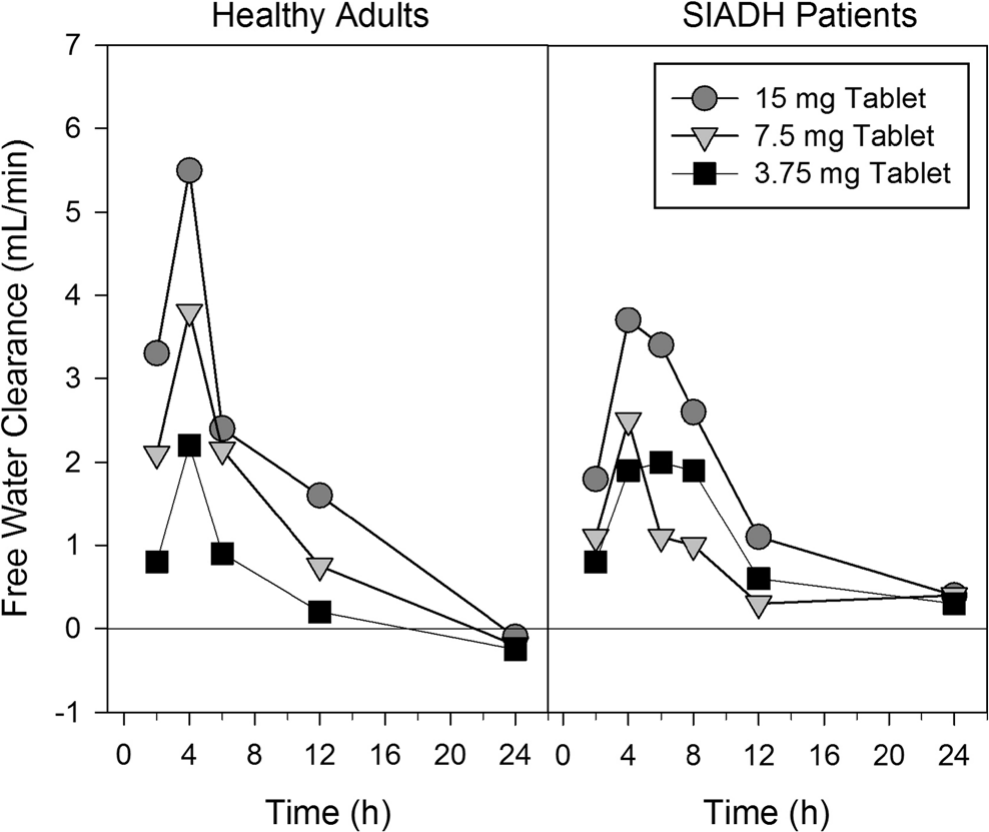
Median free water clearance plotted at the end time of the collection interval following single oral doses of tolvaptan to healthy adults and SIADH patients with hyponatremia

**Fig. 3:**
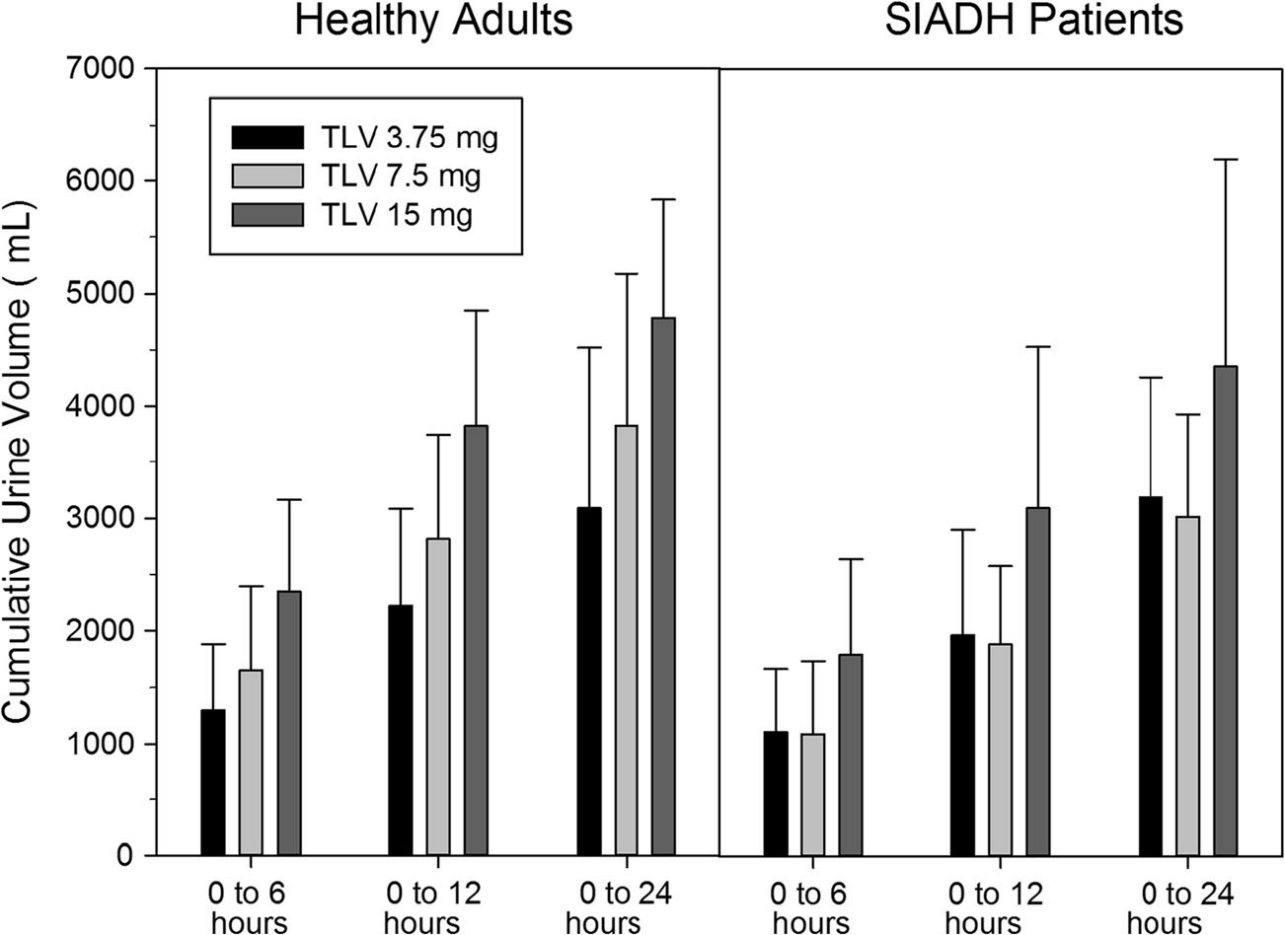
Mean (SD) cumulative urine volume following single oral doses of tolvaptan to healthy adults and SIADH patients with hyponatremia

In healthy adults, dose-dependent increases in median free water clearance (Fig. 2) and mean cumulative urine volume (Fig. 3) were observed, but in SIADH patients, 3.75- and 7.5-mg doses produced similar effects at a level similar to the 3.75-mg dose in healthy adults. To improve the clarity of the presentation, no error bars are presented in Fig. 2; coefficients of variation ranged from ~60 to 100% for values from 2 to 8 h post-dose. For 7.5- and 15-mg doses, maximal responses in free water clearance were lower in SIADH patients compared to healthy subjects. In healthy adults, the effects of tolvaptan on free water clearance lasted about 6 h for the 3.75-mg dose, as the average free water clearance for the 6 to 12 h interval was close to zero, and about 12 h for the 15-mg dose. The duration of increases in free water clearance was consistent with results from a previous trial where the minimally effective tolvaptan plasma concentrations for increasing urine output were found to be ~18 to 25 ng/mL [7]. Effects seemed to last longer in SIADH patients as mean free water clearance values were still slightly greater than zero for all doses for the 12- to 24-h interval.

**Fig. 4:**
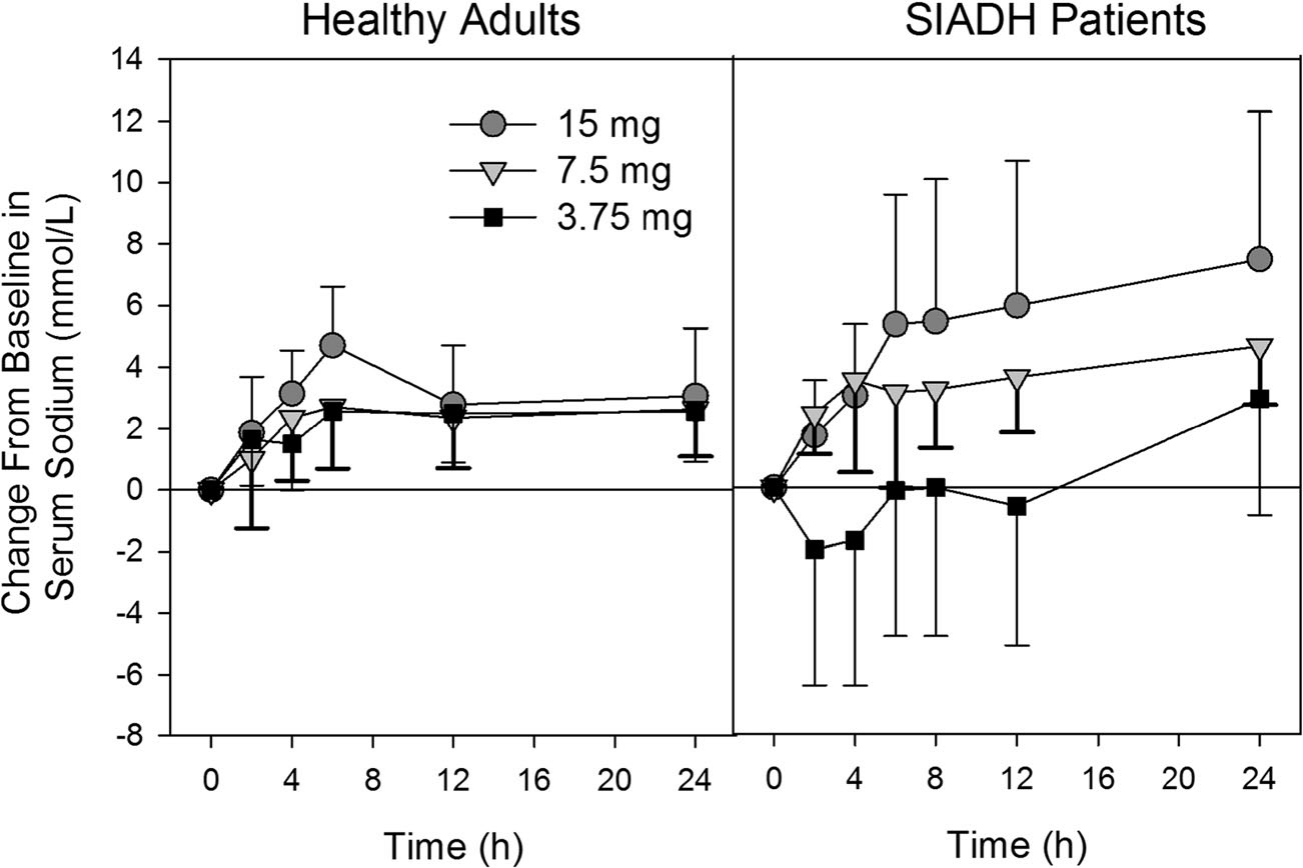
Mean (SD) change from baseline in serum sodium concentrations following single oral doses of tolvaptan to healthy adults and SIADH patients with hyponatremia

The pattern of change in serum sodium concentrations is very different for healthy adults compared to SIADH patients (Fig. 4). As summarized in Table 3, maximal increases in serum sodium in adult subjects occurred mostly earlier (median time 6 h all doses) compared to SIADH patients (median times about 24, 6, and 24 h for 3.75-, 7.5-, and 15-mg doses, respectively). Following 7.5- and 15-mg doses, increases in serum sodium in healthy adults were lower when compared to SIADH patients, but the maximal increase observed following the 3.75-mg dose appeared to be similar. In SIADH patients, no overly rapid correction of serum sodium was observed. Rapid corrections of serum sodium (defined as increase of ≥8 mmol/L in the first 8 h or ≥12 mmol/L within the first 24 h) were observed in 4 of 28 (14.3%) subjects with measurable tolvaptan concentrations. In the 3.75- and 7.5-mg treatment groups, one subject each met the 8-mmol/L criteria. In the 15-mg treatment group, one subject met the 8-mmol/L criteria and then progressed to meet the 12-mmol/L criteria and another subject met the 12-mmol/L criteria. Importantly, four (40%) subjects in the 3.75-mg dose group had clinically significant decreases of serum sodium (change of ≤−4 mmol/L in 4 h) compared to no subjects in the 7.5- and 15-mg dose groups; serum sodium decreases of 5, 6, 10, and 11 mmol/L were observed in the first 4 h with concentrations at 24 h post-dose being within ±1 mmol/L of the predose value. No rapid increases or clinically significant decreases in serum sodium were observed in healthy adults.

**Table 3:** Summary of maximal increase in serum sodium (mmol/L) following single oral doses of tolvaptan to healthy adults and SIADH patients with hyponatremia

**Parameter**	**Healthy Adults**			**SIADH Patients**		
	**3.75 mg (** ***N*** **= 14)**	**7.5 mg (** ***N*** **= 14)**	**15 mg (** ***N*** **= 14)**	**3.75 mg (** ***N*** **= 10)**	**7.5 mg (** ***N*** **= 10)**	**15 mg (** ***N*** **= 7)**
Baseline^a^	136.4 (1.8)	137.1 (2.0)	138.1 (2.0)	131.2 (6.2)	129.7 (3.8)	127.9 (4.7)
Concentration at maximal increase	139.9 (1.1)	140.6 (1.5)	143.1 (2.0)	134.8 (4.6)	135.0 (4.0)	136.6 (4.0)^b^
Maximal increase	3.5 (1.5) [3.5]	3.5 (1.7) [3.0]	5.0 (1.7) [4.5]	3.6 (3.3) [2.5]	5.3 (2.5) [6.0]	7.9 (5.3) [5.0]
Time of maximal increase (h)^c^	6 (2–24)	6 (4–24)	6 (2–24)	24.13 (2.03–24.25)	6.38 (2.05–24.28)	24.00 (6.25–24.30)

Sodium concentrations presented as mean (standard deviation [SD]), change is presented as mean (SD) [median], and time presented as median (range)

^a^Baseline was defined as predose evaluation prior to administration of tolvaptan

^b^ *N* = 8

^c^Time values for healthy adults obtained from a nominal time analysis

**Fig. 5:**
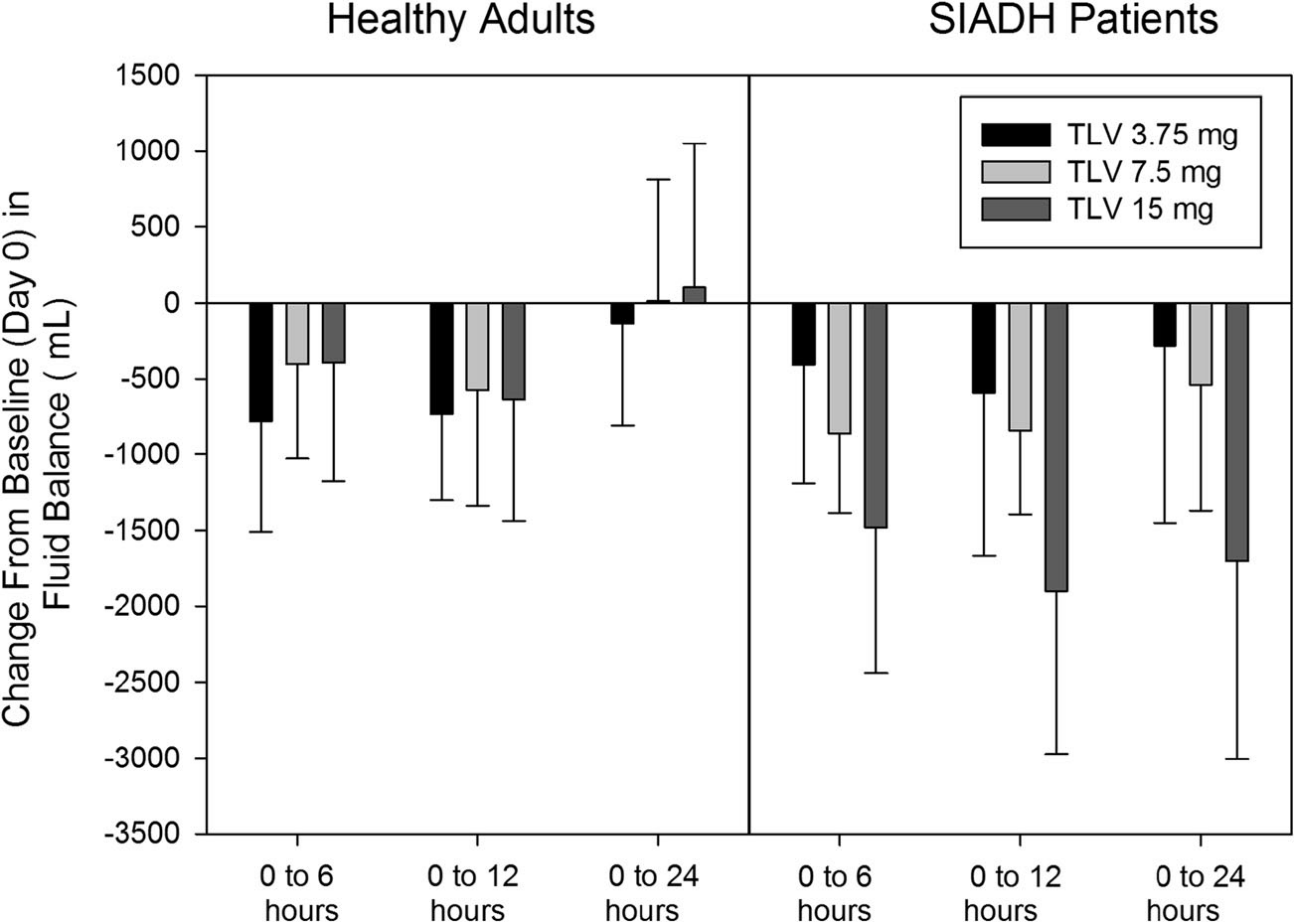
Mean (SD) change from baseline in fluid balance following single oral doses of tolvaptan to healthy adults and SIADH patients with hyponatremia

Despite greater urine output and free water clearance in healthy adults following the 7.5- and 15-mg doses, the maximal increases in serum sodium were lower compared to SIADH patients. The most likely explanation is the difference in fluid intake between the two groups (Table 4) and the resulting differences in fluid balances (Fig. 5). Healthy adults drank more water and consequently their mean fluid balances for each dose did not drop lower than −1000 mL; by 24 h post-dose, mean fluid balances were close to zero for all doses. In SIADH patients, fluid intake was lower and mean fluid balances for the 0 to 6- and 0 to 12-h periods were more negative, particularly for the 15-mg dose group, when compared to healthy adults. During the 12- to 24-h period for all doses, mean fluid balances in SIADH patients were only improved 200 to 300 mL, allowing mean serum sodium concentrations to rise during the nighttime period.

**Table 4:** Mean (SD) fluid intake following single oral doses of tolvaptan to healthy adults and SIADH patients with hyponatremia

**Tolvaptan dose**	**Collection interval**	**Mean (SD) fluid intake (mL)**	
		**Healthy adults**	**SIADH patients**
3.75 mg	0 to 6 h	1111 (589)	1036 (373)
	0 to 12 h	2167 (849)	1961 (561)
	0 to 24 h	3092 (1026)	2395 (747)
7.5 mg	0 to 6 h	1457 (663)	963 (458)
	0 to 12 h	2825 (795)	1943 (736)
	0 to 24 h	3951 (1174)	2578 (1002)
15 mg	0 to 6 h	1777 (734)	1168 (366)
	0 to 12 h	3679 (944)	2414 (828)
	0 to 24 h	4756 (1322)	3110 (1066)

*N* = 14 for healthy adults. *N* = 10, 10, and 8 for 3.75, 7.5, and 15 mg doses, respectively, to SIADH patients

### Safety

In healthy adults, 5 of 14 subjects (35.7%) administered 15 mg reported 9 treatment-emergent adverse events (TEAEs): pollakiuria was reported by 4 subjects (28.6%) and dry mouth and thirst were each reported by 2 subjects (14.3%). Three of 14 subjects (21.4%) administered 7.5 mg experienced 4 TEAEs which included diarrhea, headache, dysuria, and pollakiuria. Three of 14 subjects (21.4%) administered 3.75 mg experienced 3 TEAEs which included diarrhea, headache, and dysuria.

In SIADH patients, no subjects in the 3.75-mg treatment group experienced a TEAE. One subject (10.0%) in the 7.5-mg treatment group and three subjects (33.3%) in the 15-mg treatment group reported four and seven TEAEs, respectively. One subject in the 15-mg dose group died in the follow-up period due to the TEAE of worsening small cell lung cancer; this subject had no measurable tolvaptan plasma concentrations following dosing. Other reported TEAEs included dizziness, insomnia, and polyuria.

No subject in either trial had a TEAE related to abnormal laboratory values, vital signs, or ECG results. None of the overly rapid corrections or clinically significant decreases in serum sodium in SIADH patients were reported as TEAEs by principal investigators. No clinically significant sequelae (e.g., dysphagia, lethargy, affective changes) were reported during the follow-up period.

## Discussion

Tolvaptan *C*_max_ and AUC_∞_ values increased linearly for the doses tested. Although tolvaptan concentrations in SIADH patients were higher compared to healthy adults for the 7.5- and 15-mg doses, the difference was within the range of trial-to-trial variability observed for 30-mg doses [7, 8, 12, 13] and most likely does not represent a clinically meaningful difference as body weight adjusted values of CL/F were similar for the two groups.

Previously, it was determined that minimally effective concentrations of tolvaptan were achieved at around 25 ng/mL [7] and maximal increases in urine output occur when tolvaptan concentrations reach >100 ng/mL [13]; for single doses of 60 to 480 mg, 0- to 12-h urine volumes were similar, indicating saturation of effect for the 60-mg dose for at least 12 h post-dose. As mean (SD) peak concentrations following a 15-mg dose had been previously reported to be 135 ± 53 ng/mL [9] and 103.5 ± 39.5 ng/mL [10], it was expected that for the dose range of 3.75 to 15 mg, a dose-dependent relationship would be observed in free water clearance and urine volumes. A dose-dependent response was seen in the healthy adult trial, most likely due to the fact that all subjects got all three doses thus reducing subject variability. In the parallel-group SIADH patient trial, responses in free water clearance and urine volume were similar following 3.75- and 7.5-mg doses. In both trials, correlation plots between urine volume from 0 to 6 h and either *C*_max_ or AUC had positive slopes but the *r* ^2^ values were <0.1 and the *p* values were not significant (data not shown). This indicates that variability in sensitivity to the drug effect rather than PK is the dominant factor driving response.

The lower peak increases in free water clearance in SIADH patients could possibly be due to lower renal function, as determined by eGFR_CKD-EPI_, when compared to healthy adults. As shown in the renal impairment trial for tolvaptan [14], subjects with creatinine clearance <60 mL/min at baseline had lower peak increases in free water clearance despite all subjects but 1 having peak concentrations >200 ng/mL from 2 to 4 h post-dose.

Two of eight subjects treated with 15 mg in the SIADH patient trial reported here had rapid corrections of serum sodium. In the phase 3 clinical trials for tolvaptan approval in the treatment of hyponatremia, where 15 mg was the starting dose, 7 and 2% of tolvaptan-treated subjects with a serum sodium <130 mmol/L exceeded protocol recommended correction limits of an increase in serum sodium greater than 8 mmol/L at approximately 8 h (the first sampling point) and an increase greater than 12 mmol/L at 24 h (the second sampling point), respectively. Approximately 1% of placebo-treated subjects with a serum sodium <130 mmol/L had a rise greater than 8 mmol/L at 8 h and no patient had a rise greater than 12 mmol/L/24 h [1].

**Fig. 6:**
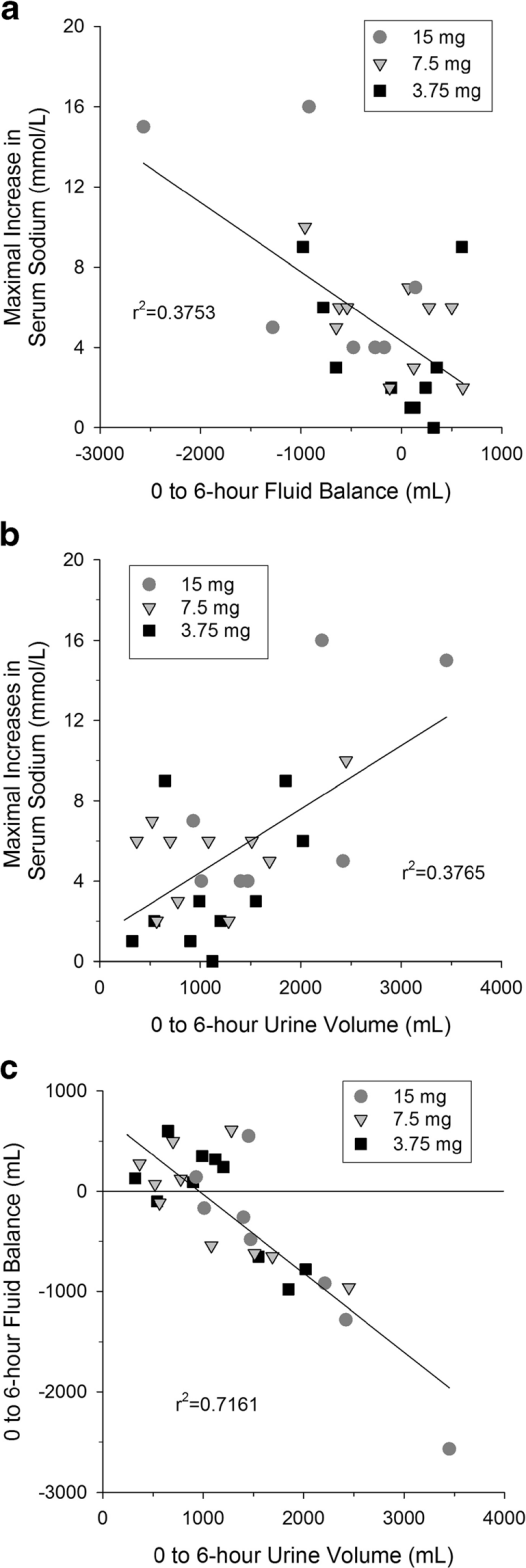
Correlations between maximal increase in serum sodium and 0- to 6-h fluid balance (**a**) and 0- to 6-h urine volume (**b**) and between fluid balance and urine volume (**c**) following single oral doses of tolvaptan to SIADH patients with hyponatremia

Results from the SIADH patient trial reported here indicate that reducing the starting dose to <15 mg will not eliminate the risk rapid corrections in serum sodium following tolvaptan administration. In order to determine if early response to tolvaptan could be an indicator of rapid correction in serum sodium, correlations (Fig. 6) between the maximal increase in serum sodium and fluid balance (panel A) or urine volume (panel B) from 0 to 6 h post-dose were investigated. The four subjects with rapid corrections had large negative fluid balances, −920, −960, −980, and −2570 mL, for 0 to 6 h post-dose. However, one subject with a fluid balance of −1280 mL had a maximal increase in serum sodium of only 5 mmol/L. The four subjects with rapid correction had urine volumes of >2000 mL for 0 to 6 h post-dose but two subjects with volumes >2000 mL had maximal increases in serum sodium of only 5 and 6 mmol/L. Because mean fluid intake in the first 6 h was similar for all doses, about 1000 mL, fluid balance was very highly correlated with urine output (Fig. 6c) with an *r* ^2^ = 0.7161.

As stated in Samsca® labeling [2], “...after initiation of treatment, patients should be closely monitored for serum sodium and volume status.” When compared to healthy subjects, SIADH patients are less likely to increase fluid intake to limit decreases in fluid balance. Limiting early, i.e., 0 to 6 h, decreases in fluid balance, when tolvaptan is having the greatest effects on free water clearance, may allow for adjustments in fluid intake that would decrease the risk of rapid correction in SIADH patients.
